# Supplementary material for: Recent decadal weakening of the summer Eurasian westerly jet attributable to anthropogenic aerosol emissions
Source: Nat Commun. 2022 Mar 3;13:1148. doi: 10.1038/s41467-022-28816-5 (PMC8894405; doi:10.1038/s41467-022-28816-5)
Supplement: Supplementary file 1 — Supplementary Information [file 41467_2022_28816_MOESM1_ESM.pdf]

Supplementary Information for manuscript

Recent decadal weakening of the summer Eurasian westerly jet attributable to anthropogenic aerosol emissions

Buwen Dong[1*], Rowan T. Sutton[1], Len Shaffrey[1] & Ben Harvey[1]

[1] National Centre for Atmospheric Science, Department of Meteorology, University of Reading

[*]corresponding author: Buwen Dong, b.dong@reading.ac.uk

Content:

- Supplementary Table 1
- Supplementary Figures 1-11

Supplementary Table 1: CMIP6 and DAMIP models used in this study

	Model	Resolution (grid number in lat×lon)	Members				Years
			All	GHG	AER	NAT	piControl
1	BCC-CSM2-MR	160×320	3	3	3	3	600
2	CanESM5	64×128	25	10	10	10	1000
3	CNRM-CM6-1	128×256	10	10	10	10	500
4	GISS-E2-1-G	90×144	10	5	5	0	850
5	HadGEM3-GC31-LL	145×192	4	4	4	4	500
6	IPSL-CM6A-LR	143×144	9	10	10	10	1000
7	MIROC6	128×256	10	3	3	3	800
8	MRI-ESM2-0	160×320	5	3	3	3	700
	All models		76	48	48	43	5950

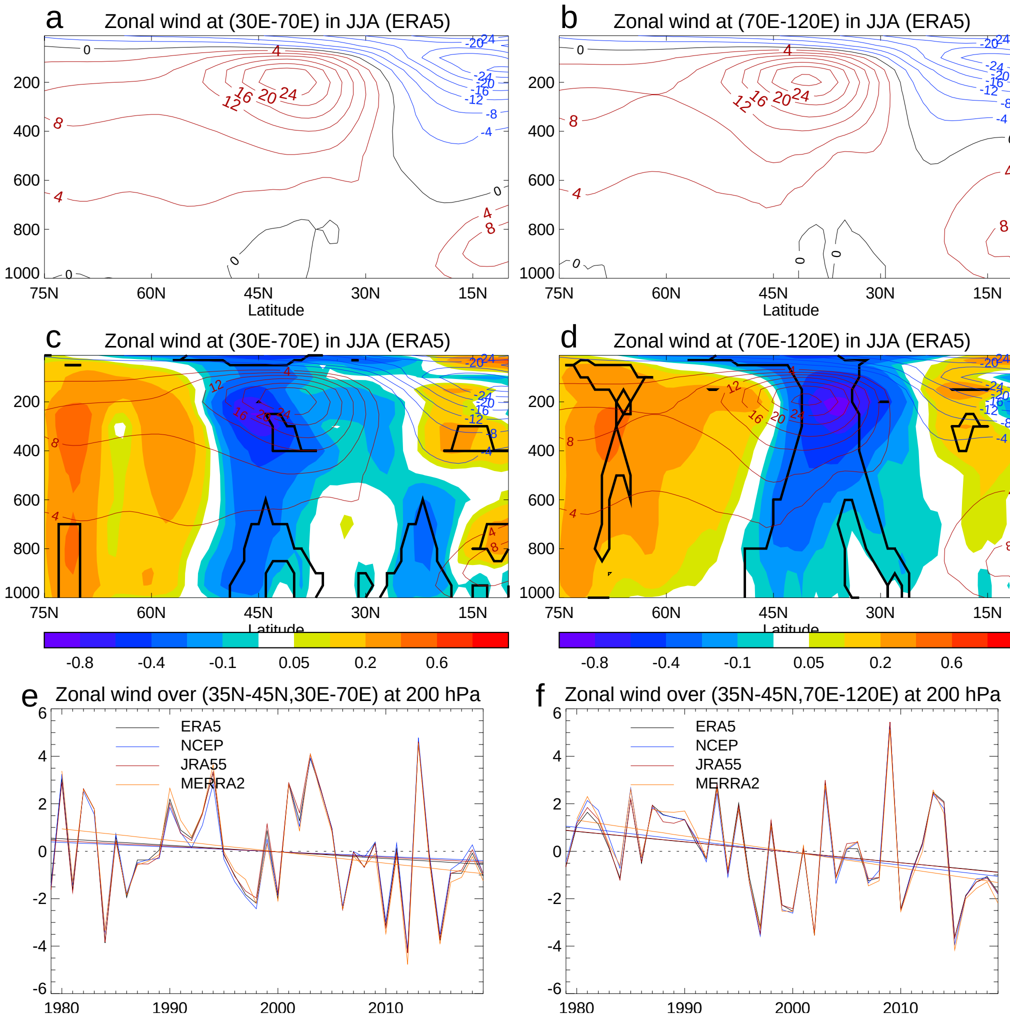

Supplementary Figure 1: The linear trends of zonal wind in summer (June, July, August) during the last four decades. a, b, climatology (m s^{-1}) (time mean over 1979-2019) at the latitude-height (hPa in pressure coordinate) cross section zonally averaged over the West Asian (30°E-70°E) and East Asian (70°E-120°E) sectors based on ERA5 reanalysis. **c, d,** linear trends (m s^{-1} decade^{-1}) with contours showing climatology. **e, f,** time series of the West Asian subtropical and East Asian subtropical westerly indices, defined as the area averaged zonal wind over the regions of 35°N-45°N, 30°E-70°E, and 35°N-45°N, 70°E-120°E at 200 hPa, based on four reanalyses and corresponding linear trends during 1979-2019 (1980-2019 for MERRA2). Thick black lines in **c, d** indicate regions where trends are statistically significant at the 10% level using the Mann-Kendall test. See Methods for details of data sets and analysis.

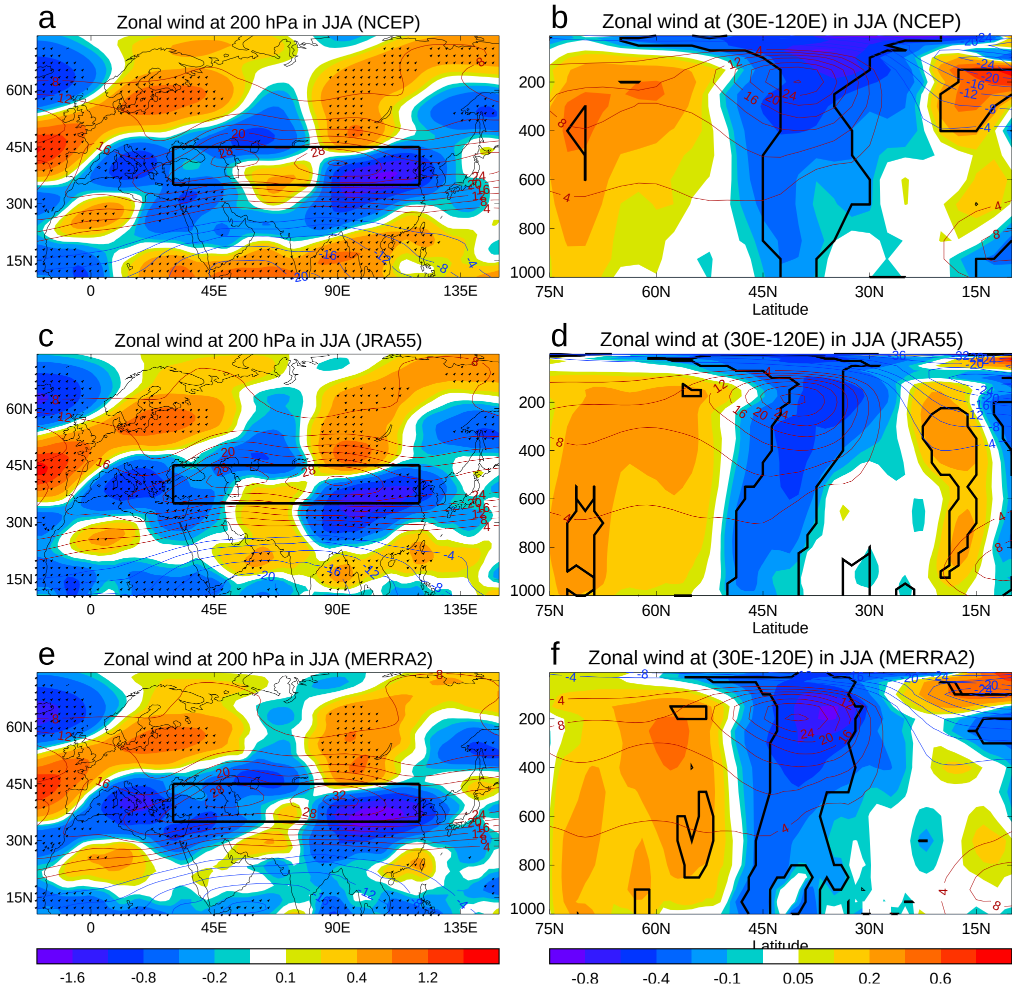

Supplementary Figure 2: The linear trends of zonal wind in summer (June, July, August) during the last four decades. a, b, linear trends (m s^{-1} $decade^{-1}$) of zonal wind at 200 hPa and at the latitude-height (hPa in pressure coordinate) cross section zonally averaged over the Eurasian sector (30°E-120°E) based on NCEP reanalysis. **c, d**, as **a, b**. but based on JRA55 reanalysis during 1979-2019. **e, f,** as **a, b,** but based on MERRA2 reanalysis during 1980-2019. Contours show the corresponding climatology (m s^{-1}). Dots in **a, c, e** and thick black lines in **b, d, f** indicate regions where trends are statistically significant at the 10% level using the Mann-Kendall test. See Methods for details of data sets and analysis.

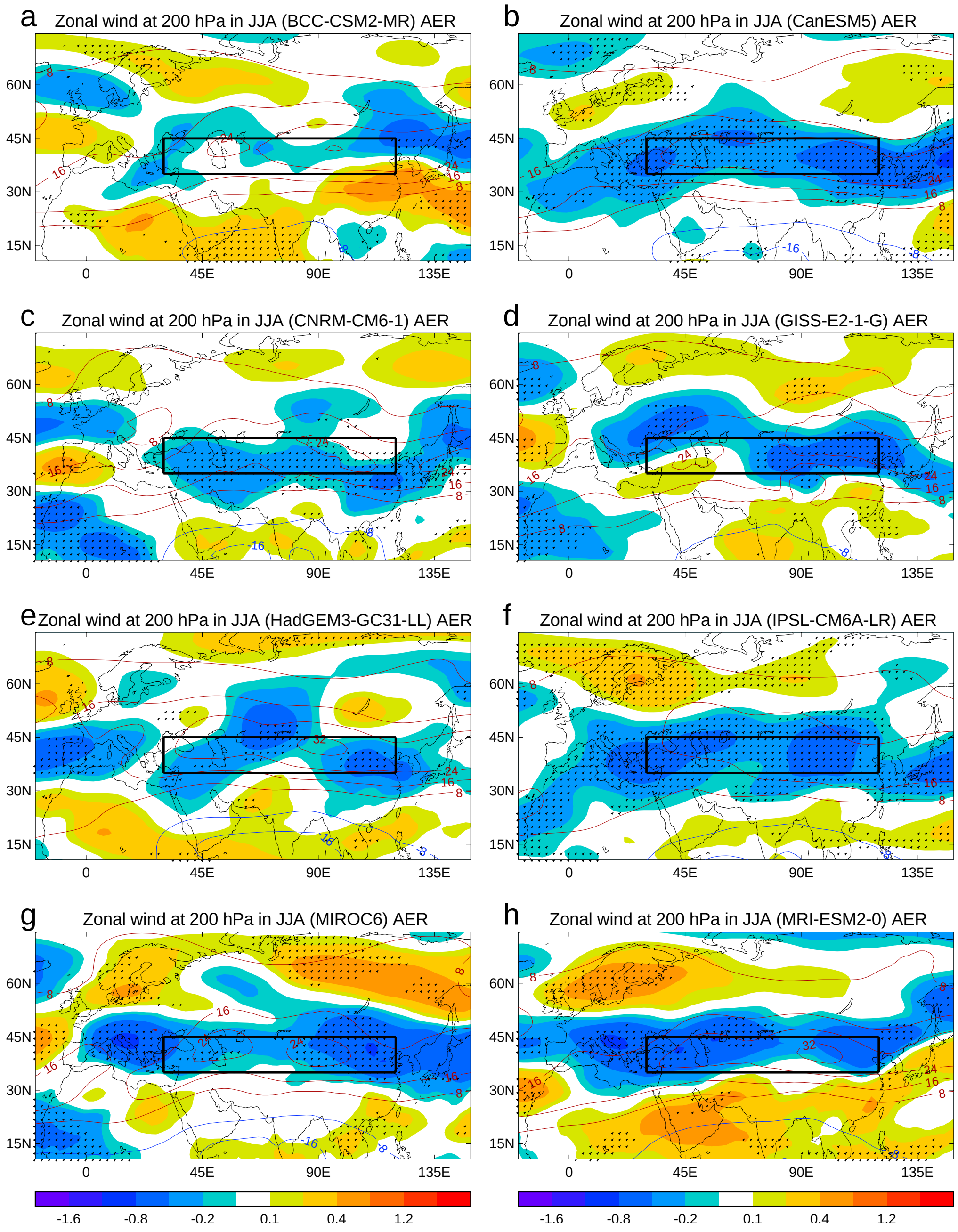

Supplementary Figure 3: The model ensemble mean linear trends of zonal wind in summer (June, July, August) at 200 hPa. a-h, linear trends (m s^{-1} decade^{-1}) during 1979-2014 for AER simulations for 8 models (Supplementary Table 1). Contours show the corresponding model climatology (m s^{-1}). Dots highlight regions where trends are statistically significant at the 10% level using the Mann-Kendall test. See Methods for details of model simulations and analysis.

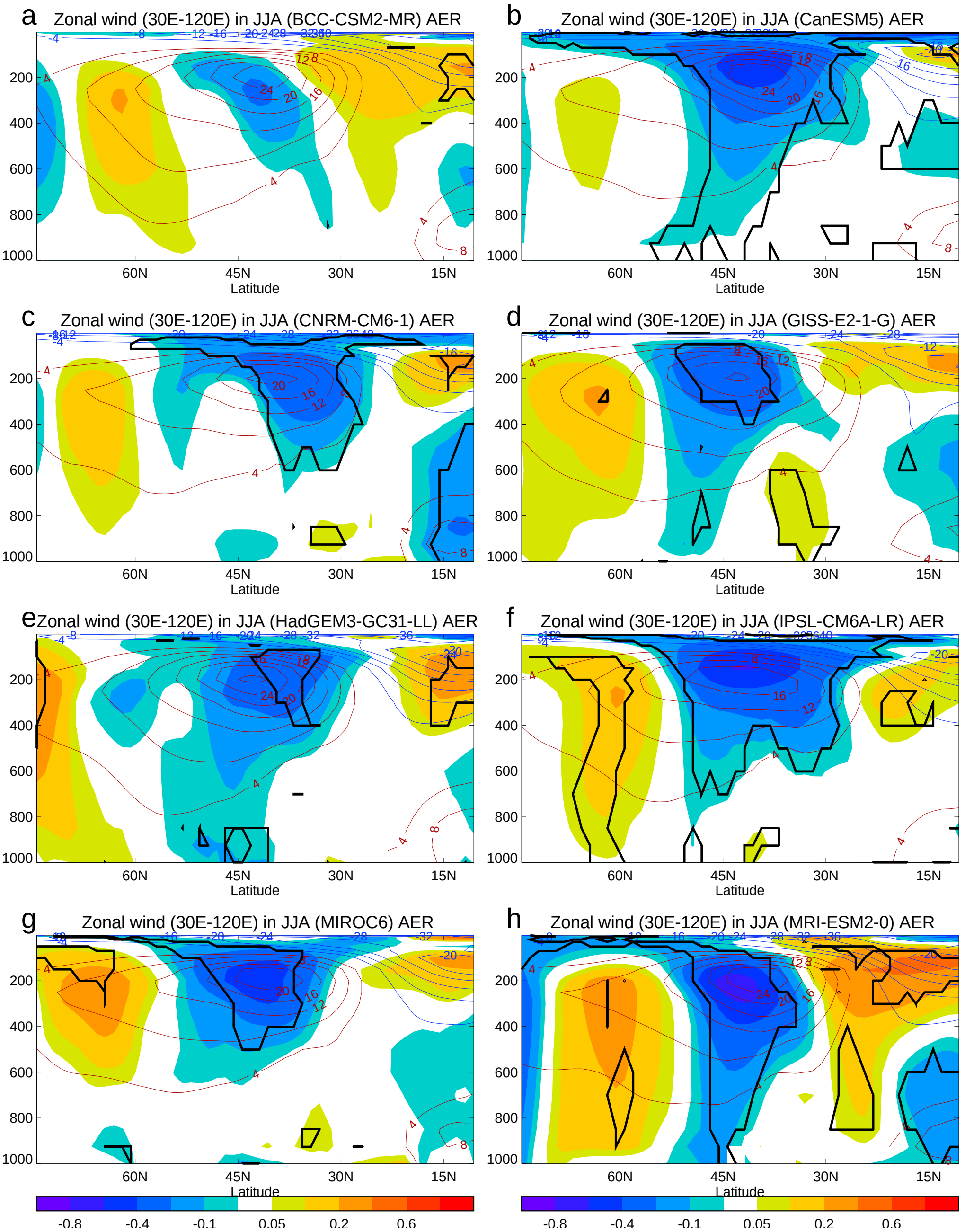

Supplementary Figure 4: The model ensemble mean linear trends of zonal wind in summer (June, July, August) at the latitude-height (hPa in pressure coordinate) cross section. a-h, linear trends (m s^{-1} $decade^{-1}$) of zonally averaged zonal wind over the Eurasian sector (30°E-120°E) during 1979-2014 for AER simulations for 8 models (Supplementary Table 1). Contours show the corresponding model climatology (m s^{-1}). Think black lines highlight regions where trends are statistically significant at the 10% level using the Mann-Kendall test. See Methods for details of model simulations and analysis.

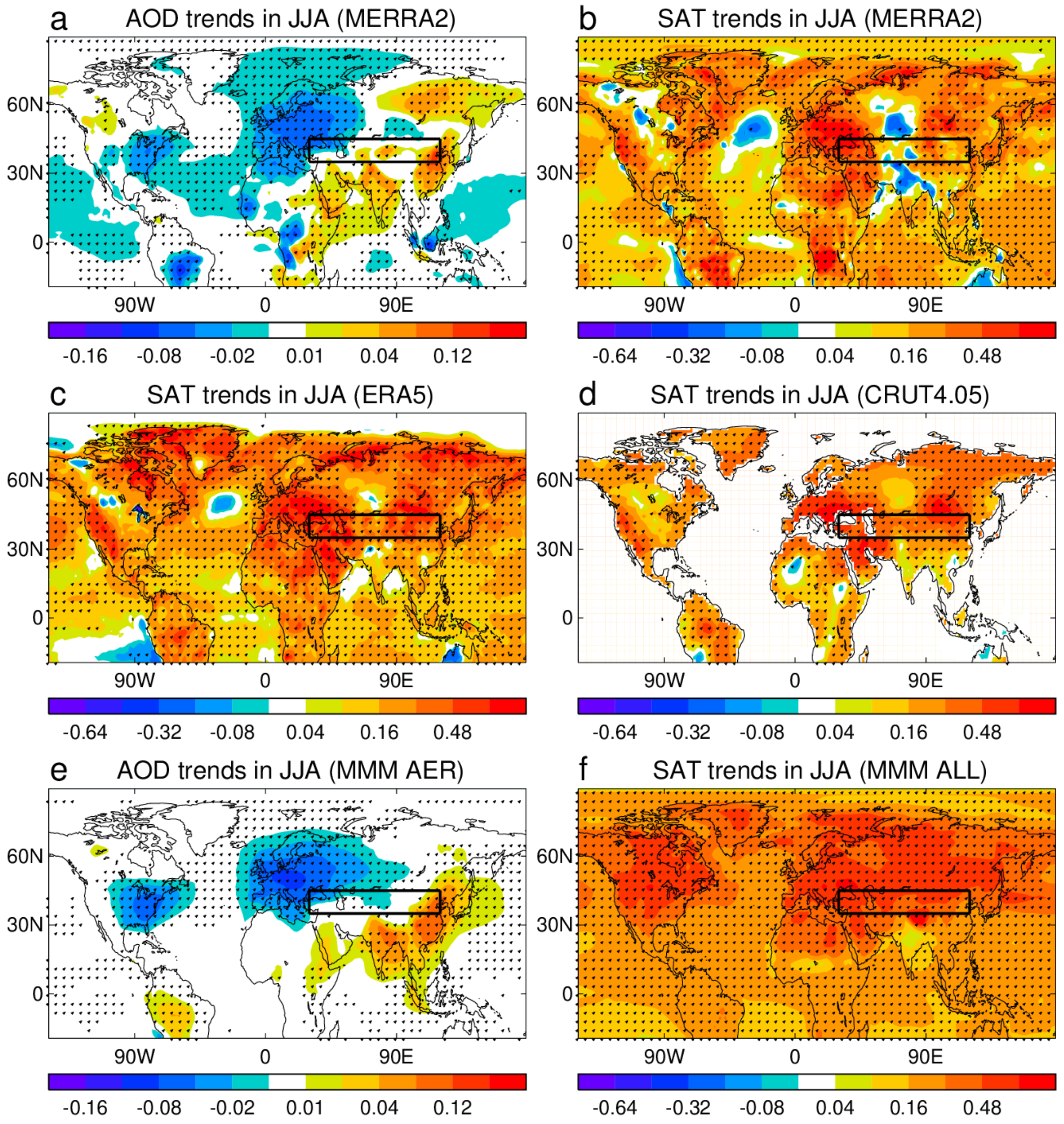

Supplementary Figure 5: The linear trends of aerosol optical depth (AOD) and surface air temperature (SAT) in summer (June, July, August) during the last four decades. a, linear trends (decade^{-1}) of AOD based on MERRA2 reanalysis during 1980-2019. **b, c, d,** linear trends of SAT (ºC decade^{-1}) based on MERRA2 (1980-2019), ERA5 and CRUT4.05 (1979-2019) data sets. **e,** linear trends (decade^{-1}) of AOD based on DAMIP AER (7 models) simulations during 1979-2014. We show AOD in AER rather than ALL simulations because more AOD data is available for AER simulations. However AOD patterns are expected to be very similar in AER and ALL. **f,** linear trends of SAT (ºC decade^{-1}) based on CMIP6 (8 models) simulations during 1979-2014**.** Dots in **a, b, c, d** indicate regions where trends are statistically significant at the 10% level using the Mann-Kendall test. Dots in **e**, **f** indicate where at least 6 (7) out of 7 (8) models have the same sign of trend. See Methods for details of data sets and analysis.

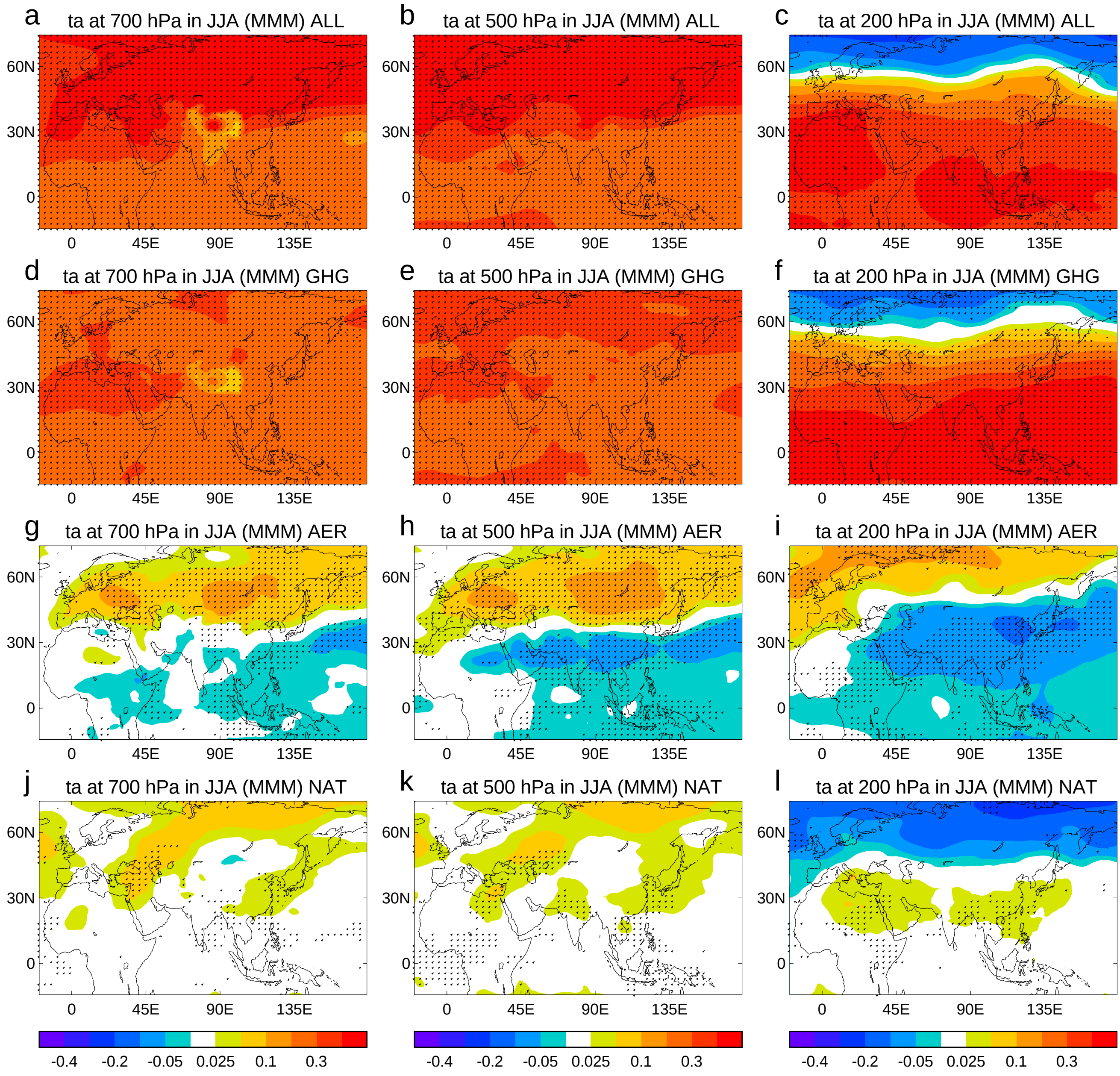

Supplementary Figure 6: Spatial patterns of multimodel mean (MMM) linear trends of temperature in summer (June, July, August). a-l, linear trends (^{o}C decade^{-1}) for CMIP6 and DAMIP simulations during 1979-2014. **a, d, g, j,** linear trends at 700 hPa). **b, e, h, k,** at 500 hPa. **c, f, i, l,** at 200 hPa. **a, b**, **c,** ALL simulations. **d, e, f,** GHG simulations. **g, h, i**, AER simulations. **j, k, l,** NAT simulations. Only 7 models are included for NAT simulations. Dots highlight regions where at least 7 (6) out of 8 (7) models showing the same sign of trends for ALL, GHG, and AER (NAT) simulations. See Methods for details of model simulations and analysis.

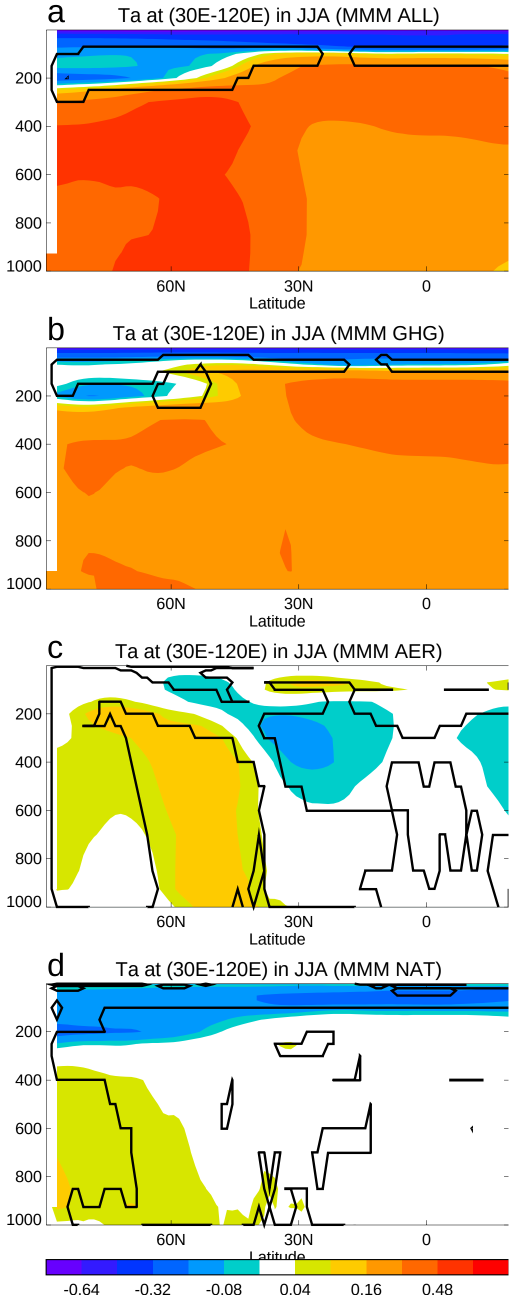

Supplementary Figure 7: Latitude-height (hPa in pressure coordinate) cross-section of multimodel mean (MMM) linear trends of temperature in summer (June, July, August). a-d, linear trends (°C decade^{-1}) of zonally averaged temperature over the Eurasian sector (30°E-120°E) during 1979-2014 for CMIP6 and DAMIP simulations. **a,** ALL simulations. **b,** GHG simulations. **c,** AER simulations. **d,** NAT simulations. Only 7 models are included for NAT simulations. Thick black lines indicate regions (large anomalies enclosed by thick black lines) where at least 7 (6) out of 8 (7) models showing the same sign of trends for ALL, GHG, and AER (NAT) simulations. See Methods for details of model simulations and analysis.

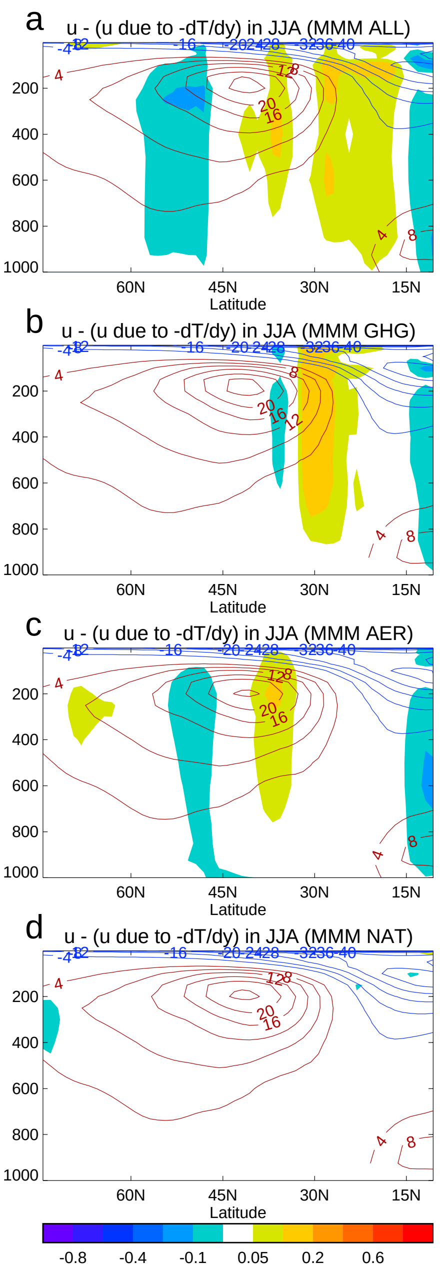

Supplementary Figure 8. The multimodel mean (MMM) residuals of zonal wind trends at the latitude-height (hPa in pressure coordinate) cross section in summer (June, July, August). Residuals of linear trends of zonal winds (m s^{-1} decade^{-1}), defined as the differences between linear trends of zonal wind (right panels of Fig. 2) and zonal winds derived from the cross-section MTG based on the thermal wind balance (right panels of Fig.5), at the latitude-height (hPa in pressure coordinate) cross section zonally averaged over the Eurasian sector (30°E-120°E) with contours showing the corresponding climatology during 1979-2014. **a,** ALL simulations. **b,** GHG simulations. **c,** AER simulations. **d,** NAT simulations. See Methods for details of model simulations and analysis.

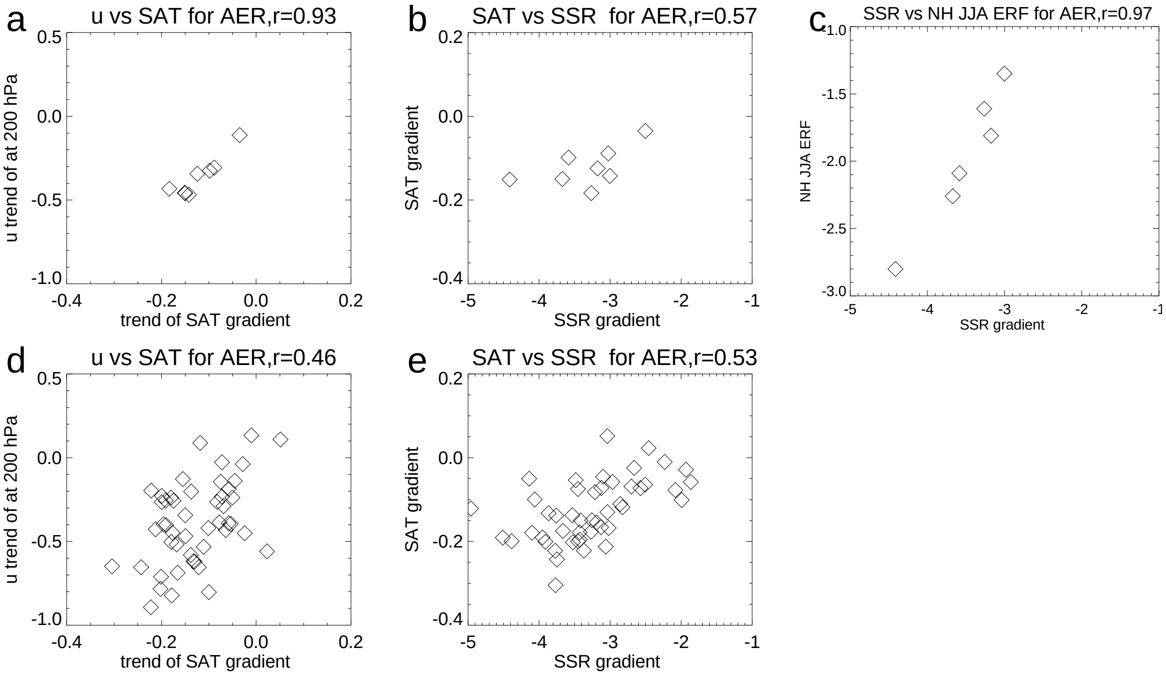

Supplementary Figure 9: Relationships of linear trends of various indices in summer (June, July, August) for DAMIP AER simulations. **a,** model ensemble mean ESWJ index (m s^{-1} decade^{-1}) vs SAT gradient index (ºC decade^{-1}). **b,** SAT gradient index vs SSR gradient index (W m^{-2} decade^{-1}). **c,** SSR gradient index vs aerosol effect radiative forcing (ERF, W m^{-2}) in summer. **d,** as **a,** but for all model members. **e,** as **b,** but for all model members. Numbers in each panel are the correlation coefficients. **c,** for six models of CanESM5, CNRM-CM6-1, GISS-E2-1-G, IPSL-CM6A-LR, MIROC6, and MRI-ESM2-0. See Methods for details of model simulations and analysis.

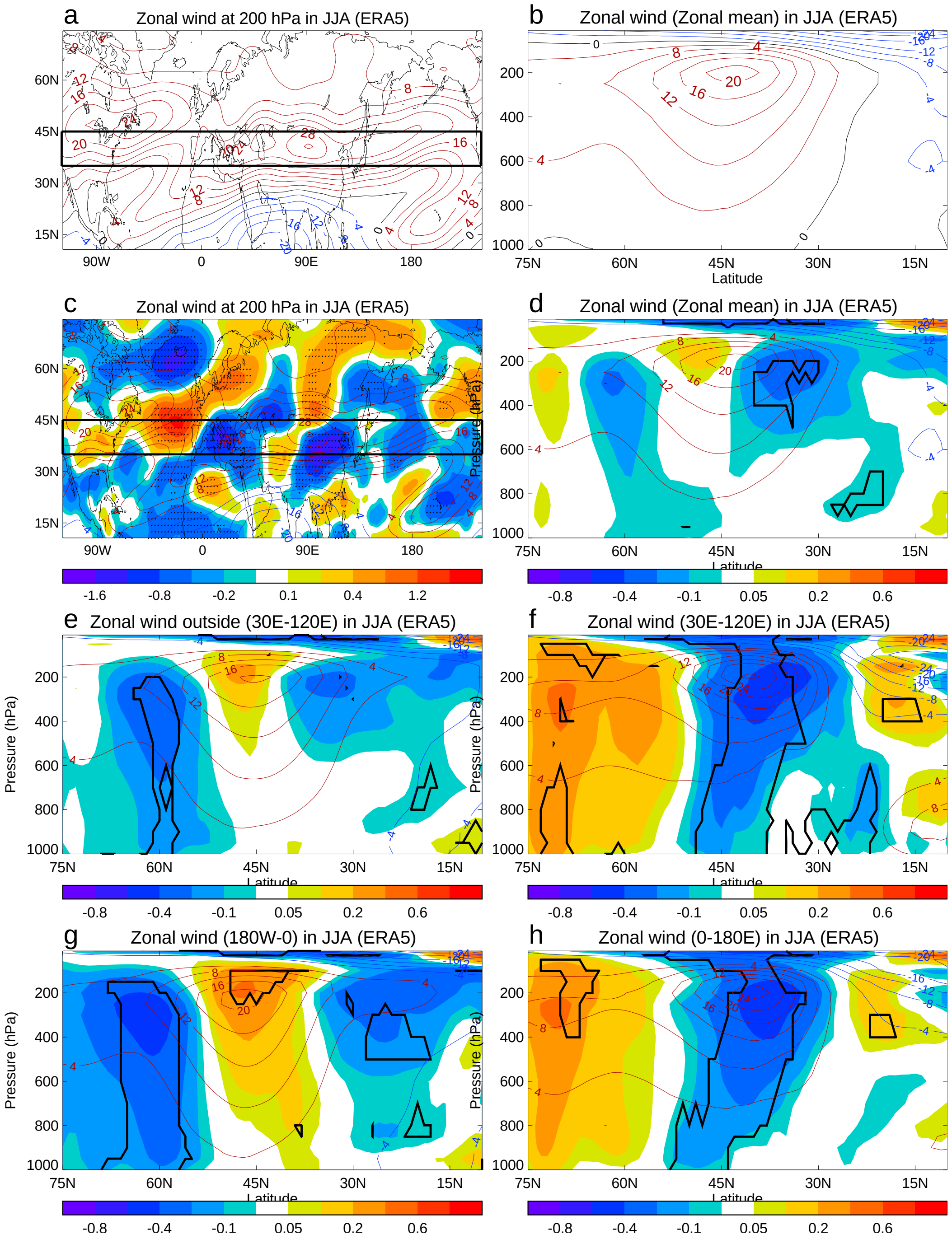

Supplementary Figure 10: The linear trends of zonal wind in summer (June, July, August) during the last four decades of 1979-2019. a, b, climatology (m s^{-1}) at 200 hPa and at the latitude-height (hPa in pressure coordinate) cross section for zonal average based on ERA5 reanalysis. **c, d,** linear trends (m s^{-1} decade^{-1}) of zonal wind at 200 hPa and at the latitude-height cross section for zonal average with contours showing climatology. **c, d,** linear trends (m s^{-1} decade^{-1}) of zonal wind at the latitude-height cross section for zonal average outside (30°E-120°E), within (30°E-120°E), (180°W-0°), and (0°-180°E), respectively, with contours showing climatology. Dots in **c** and thick black lines in **d, e, f, g, h** indicate regions where trends are statistically significant at the 10% level using the Mann-Kendall test. See Methods for details of data sets.

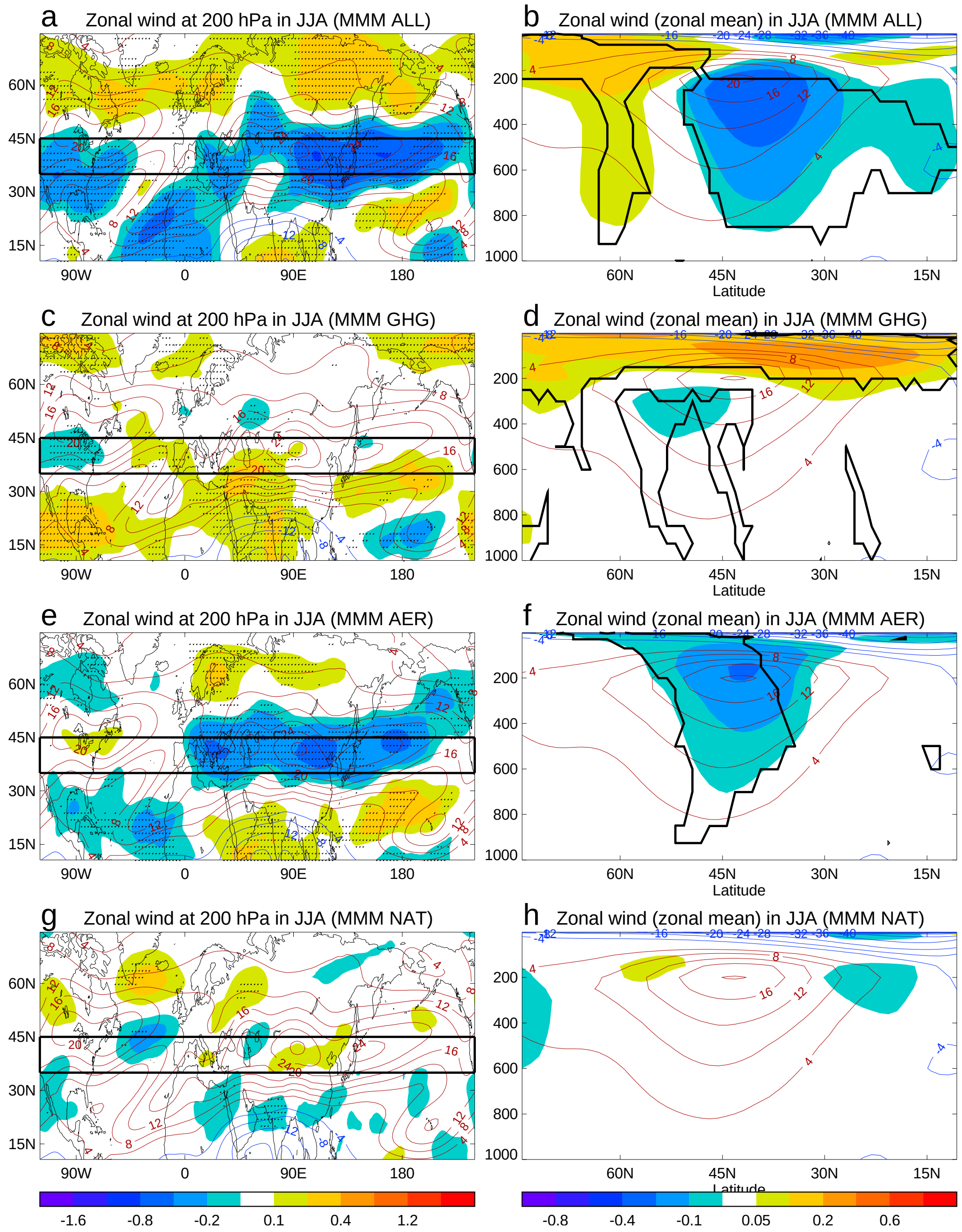

Supplementary Figure 11: The multimodel mean (MMM) linear trends of zonal wind in summer (June, July, August) during 1979-2014 in CMIP6 (DAMIP) simulations. a, c, e, g, linear trends (m s^{-1} decade^{-1}) at 200 hPa. **b, d, f, h**, linear trends (m s^{-1} decade^{-1}) of zonally averaged zonal wind at the latitude-height (hPa in pressure coordinate) cross section. Contours show the corresponding climatology and black boxes (left) highlight the latitude band (35°N-45°N). **a, b**, ALL simulations. **c, d,** GHG simulations. **e, f,** AER simulations. **g, h,** NAT simulations. Only 7 models are included in panels **g** and **h** for NAT simulations. Dots (left) and thick black lines (right) highlight regions where at least 7 (6) out of 8 (7) models showing the same sign of trends for ALL, GHG, and AER (NAT) simulations. See Methods for details of model simulations and analysis.
